# Salvage Surgery for Primary Renal Pelvic Urothelial Carcinoma After Enfortumab Vedotin: A Case of Durable Remission With Nectin‐4 Loss

**DOI:** 10.1002/iju5.70122

**Published:** 2025-12-12

**Authors:** Takahito Wakamiya, Yuya Iwahashi, Hiroki Kawabata, Satoshi Muraoka, Shimpei Yamashita, Fumiyoshi Kojima, Yasuo Kohjimoto

**Affiliations:** ^1^ Department of Urology Wakayama Medical University Wakayama Japan; ^2^ Department of Human Pathology Wakayama Medical University Wakayama Japan

**Keywords:** antibody‐drug conjugates, chemotherapy, renal pelvis, salvage therapy, urothelial carcinoma

## Abstract

**Introduction:**

Enfortumab vedotin is a standard therapy for advanced urothelial carcinoma, but its efficacy may be limited by acquired resistance and spatial heterogeneity of Nectin‐4 expression.

**Case Presentation:**

We report the case of a 76‐year‐old woman with cT3N2M0 left renal pelvic urothelial carcinoma that was treated with first‐line chemotherapy, followed by pembrolizumab, and subsequently enfortumab vedotin. After 16 cycles of enfortumab vedotin, lymph node metastases remained shrunk, but isolated progression of the primary tumor occurred. Salvage nephroureterectomy was therefore performed, and the patient has remained recurrence‐free for one year postoperatively. Immunohistochemistry showed a marked decline in Nectin‐4 expression in the resected tumor compared with at the time of the initial biopsy.

**Conclusion:**

This case highlights the potential role of surgery for localized progression during enfortumab vedotin therapy. Nectin‐4 reassessment may be considered selectively at decision‐changing junctures when results are likely to alter management.

## Keynote Message

Our case suggests the efficacy of surgical intervention in managing localized progression during enfortumab vedotin therapy. In practice, targeted biopsy at decision‐changing junctures may better inform management for advanced urothelial carcinoma.

**Abbreviations**
CT: computed tomography
EV: enfortumab vedotin
H‐score: histologic score

## 1 Introduction

Enfortumab vedotin (EV) is a standard treatment for patients with advanced urothelial carcinoma in whom there has been progression after platinum‐based chemotherapy and immune checkpoint inhibitors. Significant response rates and survival benefits with EV have been demonstrated in clinical trials compared with standard chemotherapy [1], and real‐world studies have further validated its efficacy and manageable safety profile [2]. Moreover, there is potential utility in combination with radiotherapy for prolonged disease control [3]. EV can induce substantial tumor shrinkage even in heavily pretreated cases, but its efficacy may be transient, which underscores the need for optimal sequencing and multimodal strategies [4]. Here, we present the case of a patient with isolated primary tumor progression during EV therapy in which salvage nephroureterectomy resulted in sustained disease control.

## 2 Case Presentation

**FIGURE 1:**
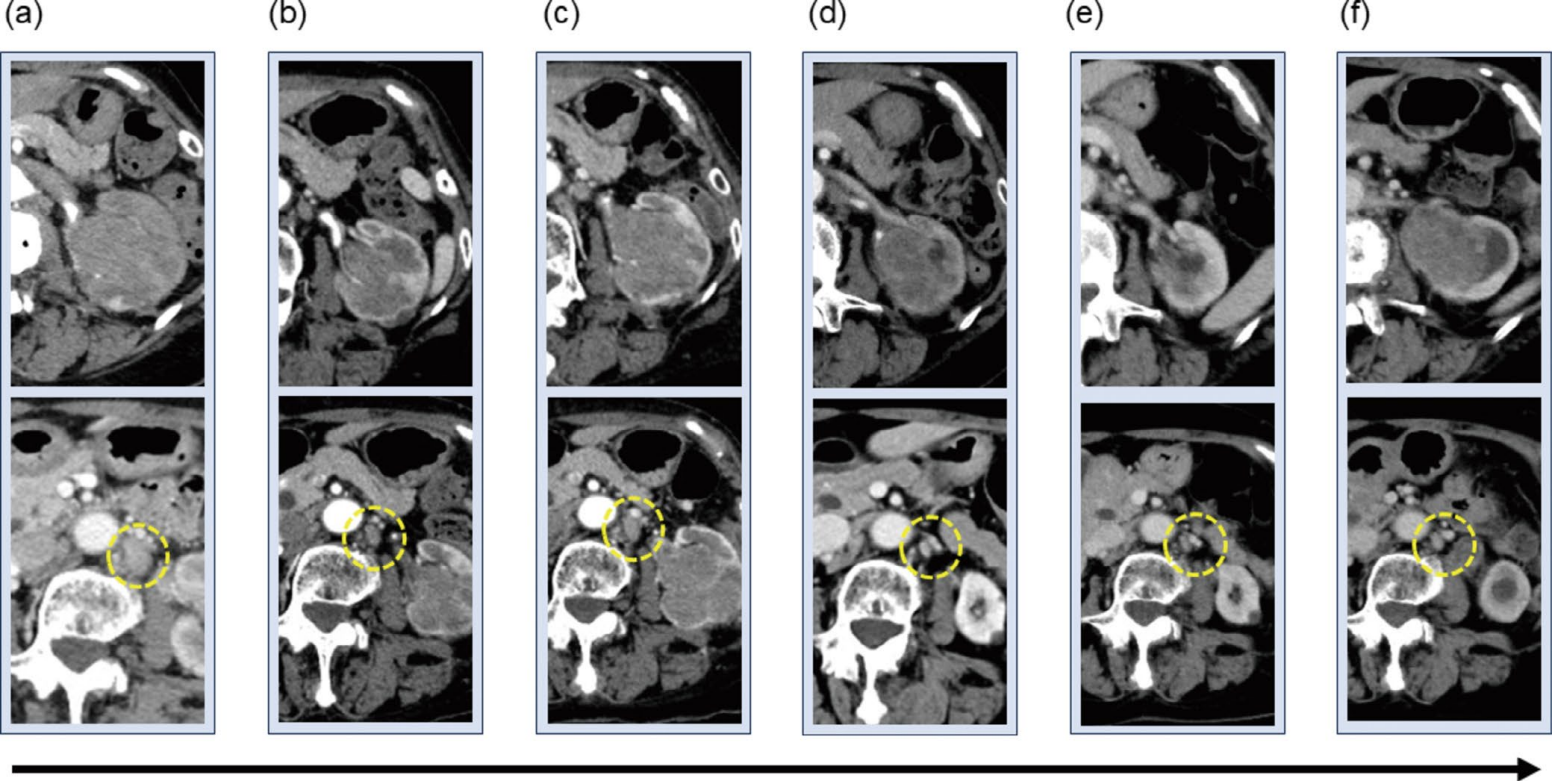
(a) CT image at diagnosis. (b) CT image after two cycles of gemcitabine and cisplatin. (c) CT image after five cycles of gemcitabine and cisplatin. (d) CT image after 11 cycles of pembrolizumab. (e) CT images after three cycles of EV following achievement of PR. (f) CT image before robot‐assisted nephroureterectomy.

**FIGURE 2:**
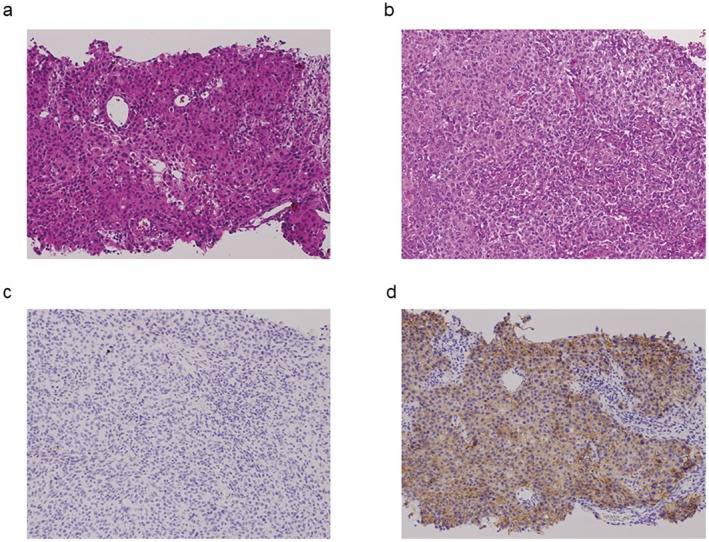
(a) Pathological image of percutaneous biopsy showing urothelial carcinoma (HE, 100×). (b) Pathological image of specimen removed by nephroureterectomy showing urothelial carcinoma (HE, 100×). (c) Pathological image of specimen removed by nephroureterectomy showing urothelial carcinoma: Nectin‐4 almost negative (Nectin‐4, 100×). (d) Pathological image of percutaneous biopsy showing urothelial carcinoma: Nectin‐4 strong positive (Nectin‐4, 100×).

A 76‐year‐old woman was referred to our Department of Urology with macroscopic hematuria. Ultrasound‐guided percutaneous biopsy of the left renal tumor revealed renal pelvic urothelial carcinoma, staged as cT3N2M0 (Figures 1a and 2a). Systemic chemotherapy with gemcitabine (100% dose) and cisplatin (75% dose) was initiated as first‐line treatment for metastatic urothelial carcinoma. After two cycles, a partial response was achieved (Figure 1b), but after five cycles we observed disease progression with enlargement of both the primary tumor and regional lymph nodes (Figure 1c). Pembrolizumab was subsequently administered as second‐line therapy. Stable disease was maintained for six cycles, but after 11 cycles, we could confirm radiological progression (Figure 1d).

**FIGURE 3:**
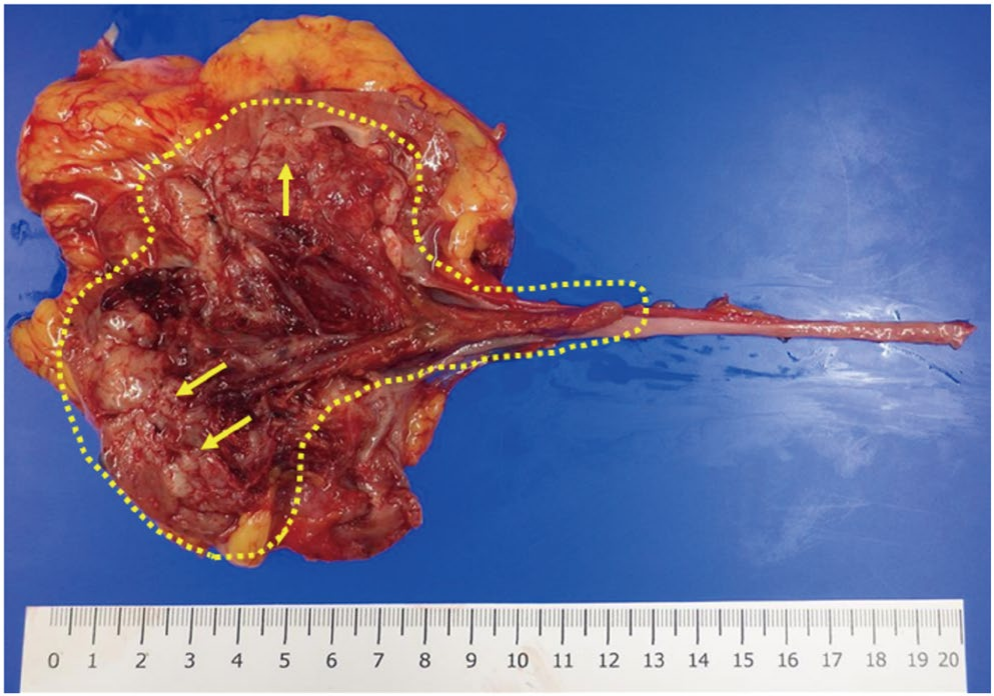
Macroscopic nephroureterectomy specimen of the left renal pelvis. The tumor, including its extension into the ureter, is delineated by a dotted outline; arrows indicate invasion into the renal parenchyma.

Subsequently, we introduced EV as third‐line therapy. Following three cycles, a partial response was achieved and tumor shrinkage was maintained thereafter (Figure 1e). After approximately one year of EV treatment (11 cycles), the lymph node metastases remained controlled, but there was progression of the primary tumor. EV therapy was continued for a total of 16 cycles. Adverse events included grade 2 alopecia, skin rash, and dysgeusia. We implemented dose reductions due to the skin rash: to 80% from the second cycle, 70% from the 10th cycle, and 60% from the 11th cycle. No new metastatic lesions developed and lymph node involvement remained stable, so we performed robot‐assisted nephroureterectomy for gradual enlargement of the primary renal pelvic tumor following 16 cycles of EV therapy (Figure 1f). Intraoperatively, mild adhesions were noted around the para‐aortic lymph nodes, but there were no adhesions in the perirenal region. Lymphadenectomy was not performed because the lymph nodes remained consistently reduced in size and because there is limited evidence supporting curative benefit from lymphadenectomy in this setting. Macroscopically, the tumor showed invasion into the renal parenchyma and partial extension into the ureter (Figure 3). Histopathological examination revealed high‐grade urothelial carcinoma, pathological stage T3 with lymphovascular invasion, and negative surgical margins (Figure 2b). Immunohistochemistry staining for Nectin‐4 in the resected tumor specimen following EV therapy demonstrated markedly reduced membranous expression, with an H‐score of 5 (Figure 2c). In contrast, the initial percutaneous biopsy specimen showed strong membranous expression, with an H‐score of 200 (Figure 2d). Following surgery, no further systemic therapy was administered. Approximately one year postoperatively, the patient has remained recurrence‐free, including in the bladder. The patient has given informed consent to the publication of her case.

## 3 Discussion

Our case had a distinct clinical course in which prolonged EV therapy achieved sustained control of lymph node metastases but progression of the primary tumor. Salvage nephroureterectomy was performed after 16 cycles of EV, leading to durable disease control. Rather than reflecting uniform resistance, this scenario suggests spatial heterogeneity in therapeutic response, which is likely influenced by differential Nectin‐4 expression. The initial biopsy showed strong Nectin‐4 expression (H‐score 200), whereas the post‐EV specimen showed markedly reduced expression (H‐score 5), indicating possible acquired resistance.

Nectin‐4 is frequently expressed in urothelial carcinoma and is utilized as the therapeutic target of EV [1]. A retrospective cohort study reported that 39% of metastatic lesions showed decreased Nectin‐4 compared to the primary tumor, which correlated with reduced EV efficacy [5]. Conversely, another retrospective study demonstrated higher expression in lymph node metastases than in primary lesions (87% vs. 63%), particularly in luminal subtypes [6]. In our patient's case, radiographic stability of lymph nodes may reflect preserved target expression, which is consistent with these reported findings. In addition, tumor phenotype may also influence treatment efficacy. Nectin‐4 expression varies across molecular subtypes of urothelial carcinoma, with higher expression in luminal subtypes than in basal subtypes [7]. Beyond target loss, multiple mechanisms can attenuate EV activity: induction of drug‐efflux transporters (ABCB1/MDR1, ABCC1/MRP1, ABCG2/BCRP) [8], alterations in microtubule dynamics and MMAE sensitivity, defects in internalization–lysosomal trafficking, and microenvironmental factors (stromal density, vascularity, inflammation) [9, 10]. These may have coexisted with the observed Nectin‐4 decline in the primary tumor. Salvage surgery being performed after EV therapy has been reported in cases with marked reduction of bladder tumor and lymph node metastasis [11], but in our case, salvage surgery was performed for localized progression despite ongoing systemic control. There has been no recurrence postoperatively, which may suggest the validity of resection of localized progressing lesions in carefully selected patients, particularly when progression is limited to the primary site and metastatic lesions remain stable without the emergence of new metastases for a few months even after progression. In the present case, we decided not to remove the lymph node metastasis. From the perspective of potential future regrowth, simultaneous resection might have been considered as an alternative strategy. Nevertheless, the metastatic lymph node had demonstrated long‐term stability and sustained regression, and we wanted to avoid the invasiveness of systematic lymphadenectomy, so we chose to limit the procedure to just resection of the primary lesion. Careful follow‐up is essential, and if nodal regrowth occurs, biopsy or surgical excision should be considered.

This case underscores the value of dynamic biomarker reassessment during EV therapy. However, routine serial biopsies at fixed intervals are not feasible in practice, so site‐selective biopsy at decision‐changing junctures (e.g., oligoprogression or localized progression) and prioritizing the progressing lesion to reassess Nectin‐4 (H‐score) and resistance markers, is thought to represent a pragmatic approach. In the absence of lymphadenectomy, histologic confirmation of nodal control was not possible in this case, but sustained radiologic stability suggested ongoing sensitivity. A key limitation of this report is that platinum‐based chemotherapy and pembrolizumab preceded EV, so a decline in Nectin‐4 expression may have begun before the initiation of EV. Moreover, the absence of serial pre‐EV assessments makes it difficult to definitively attribute the observed changes to EV exposure.

In conclusion, this case highlights the potential role of surgery for localized progression during EV. Nectin‐4 reassessment may be considered selectively at decision‐changing junctures when results are likely to alter management.

## Consent

The patient has provided informed consent to publication of the details of her case and use of the images.

## Conflicts of Interest

Yasuo Kohjimoto is both an Editorial Board member of the *International Journal of Urology Case Reports* and a co‐author of this article. To minimize bias, they were excluded from all editorial decision‐making related to the acceptance of this article for publication. The other authors declare no conflicts of interest.

## Data Availability

Research data are not shared.
